# Semantic Segmentation of Pancreatic Cancer in Endoscopic Ultrasound Images Using Deep Learning Approach

**DOI:** 10.3390/cancers14205111

**Published:** 2022-10-18

**Authors:** Kangwon Seo, Jung-Hyun Lim, Jeongwung Seo, Leang Sim Nguon, Hongeun Yoon, Jin-Seok Park, Suhyun Park

**Affiliations:** 1 Department of Electrical and Electronics Engineering, Chung-Ang University, Seoul 06974, Korea; 2 Division of Gastroenterology, Department of Internal Medicine, Inha University School of Medicine, Incheon 22332, Korea; 3 Department of Electronic and Electrical Engineering, Ewha Womans University, Seoul 03760, Korea

**Keywords:** endoscopic ultrasonography, pancreatic cancer, surgical therapy, segmentation, deep learning

## Abstract

**Simple Summary:**

Surgical therapy is critical to pancreatic cancer survival. The segmentation of pancreatic cancer in endoscopic ultrasonography (EUS) images can provide critical characteristics of the pancreatic cancer for surgical therapy. However, EUS has high operator dependency, and it requires a considerable level of experience and competency to stage pancreatic cancer. Deep learning approaches have been used on EUS images, but there have been no studies on the segmentation of pancreatic cancer. The purpose of this study is to segment pancreatic cancer from EUS images using a neural network model with deep attention features, called DAF-Net. The significance of this study lies in the successful segmentation performance of the pancreatic cancer using the DAF-Net, regardless of the size and location of the cancer, and its usefulness in preoperative planning.

**Abstract:**

Endoscopic ultrasonography (EUS) plays an important role in diagnosing pancreatic cancer. Surgical therapy is critical to pancreatic cancer survival and can be planned properly, with the characteristics of the target cancer determined. The physical characteristics of the pancreatic cancer, such as size, location, and shape, can be determined by semantic segmentation of EUS images. This study proposes a deep learning approach for the segmentation of pancreatic cancer in EUS images. EUS images were acquired from 150 patients diagnosed with pancreatic cancer. A network with deep attention features (DAF-Net) is proposed for pancreatic cancer segmentation using EUS images. The performance of the deep learning models (U-Net, Attention U-Net, and DAF-Net) was evaluated by 5-fold cross-validation. For the evaluation metrics, the Dice similarity coefficient (DSC), intersection over union (IoU), receiver operating characteristic (ROC) curve, and area under the curve (AUC) were chosen. Statistical analysis was performed for different stages and locations of the cancer. DAF-Net demonstrated superior segmentation performance for the DSC, IoU, AUC, sensitivity, specificity, and precision with scores of 82.8%, 72.3%, 92.7%, 89.0%, 98.1%, and 85.1%, respectively. The proposed deep learning approach can provide accurate segmentation of pancreatic cancer in EUS images and can effectively assist in the planning of surgical therapies.

## 1. Introduction

The incidence and mortality rates of pancreatic cancer are growing quickly (doubling in 10 years from 1997) due to the aging of the global population [1], as 90% of the population diagnosed with pancreatic cancer are older than 55. The prognosis of pancreatic cancer is critical given the 5-year survival rate for pancreatic cancer is approximately 6% [2]. Pancreatic cancer is a highly invasive malignancy as it can quickly invade surrounding tissues and organs [1,3]. Due to the location and nonspecific symptoms of pancreatic cancer for diagnosis, there are difficulties in detecting pancreatic cancer. To diagnose pancreatic cancer, multi-detector computed tomography (MDCT), magnetic resonance imaging (MRI), and endoscopic ultrasonography (EUS) are widely used imaging methods [2,3,4,5,6]. EUS is an ultrasound imaging technology equipped with a high-frequency transducer at the end of the endoscope. The endoscopic transducer is placed in the proximal stomach to obtain high-resolution images of the pancreas [5,7]. Endoscopic ultrasonography-guided fine-needle aspiration (EUS-FNA) is a reliable diagnostic tool for pancreatic cancer [3,5]. Surgical therapy is a critical factor in surviving pancreatic cancer. EUS can stage pancreatic cancer and plays an important role in finding surgically resectable lesions. Semantic segmentation of the cancer area from EUS images is expected to provide the physical characteristics of the cancer, such as size, location, and shape, to help determine the plan for surgical therapies.

Deep learning models are being developed to extract complex features from image data and are widely used in the field of medical imaging [8]. Previous studies have used deep learning models for the classification and segmentation of pancreatic EUS images [9,10,11,12,13]. Kuwahara et al. [9] proved that a deep learning model (ResNet50) can diagnose the malignancy of Intraductal Papillary Mucinous Neoplasm (IPMN). Our previous study classified two types of pancreatic cyst lesions, mucinous cystic neoplasm (MCN) and serous cystic neoplasm (SCN), through Resnet50 [10]. Zhang et al. [11] conducted classification and segmentation to locate the regions of the pancreas through deep learning models (Resnet50 and U-Net++, respectively). Iwasa et al. [12] performed pancreatic cancer segmentation using contrast-enhanced EUS (CE-EUS) images through a deep learning model (U-Net). Additionally, Oh et al. [13] evaluated the segmentation performance of pancreatic cyst lesions through the application of various deep learning models (U-Net, Attention U-Net (Att-U-Net), U-Net++, and Residual U-Net) to EUS images.

In this study, we aim to suggest an efficient deep learning model for pancreatic cancer segmentation by comparing several deep learning models trained by EUS images acquired from patients diagnosed with pancreatic cancer. While recent studies [9,10,11,12,13] have employed deep learning approaches and demonstrated good performance for various other purposes using pancreatic EUS images, to the best of our knowledge, this is the first study on pancreatic cancer segmentation using EUS images. The segmentation performance of the suggested network was evaluated for different T stages [14] and locations of the pancreatic cancer.

## 2. Materials and Methods

### 2.1. Data Acquisition

EUS data were collected between January 2010 and July 2021 at the endoscopic center of the Inha University Hospital. This study was reviewed and approved by the Institutional Review Boards of the Inha University Hospital (2020-12-005). Patients were sedated intravenously with propofol (20–120 mg) and midazolam (2.0–5.0 mg) after 8 hours of fasting. EUS was performed using linear (GF-UCT 240; Olympus Optical corp. Ltd., Tokyo, Japan) echoendoscopes with an ultrasound scanning system (SSD 5500, 5 and 10; Aloka, Tokyo, Japan). Video recording started from the point when the echoendoscope entered the stomach and reached the pancreas, and the recording was terminated when all procedures were completed. The procedure was conducted by endosonographers with more than 5 years of experience. EUS images from patients diagnosed with pancreatic cancer were captured from the video recordings. For this study, 330 EUS images were acquired from 150 patients. A summary of the characteristics of the patients (age and gender) and the cancer (size, T-stage, and location) is shown in Table 1.

The operator performed the procedure after thoroughly reviewing clinical data such as MDCT, positron emission tomography (PET), or tumor markers (CA19-9). For the patients with a suspected pancreatic cancer diagnosis, EUS-guided fine-needle aspiration (FNA) was performed when there were signs of pancreatic cancer observed in the EUS images, such as a heterogeneous hypoechoic mass with irregular margins, tumorous pseudopodia, or the abrupt narrowing of the upstream pancreatic duct. In the case in which the results of the EUS-FNA were atypical or negative for malignancy, the patient underwent surgical treatment with further confirmation of a cancer diagnosis provided by a biopsy.

### 2.2. Method Overview

**Figure 1:**
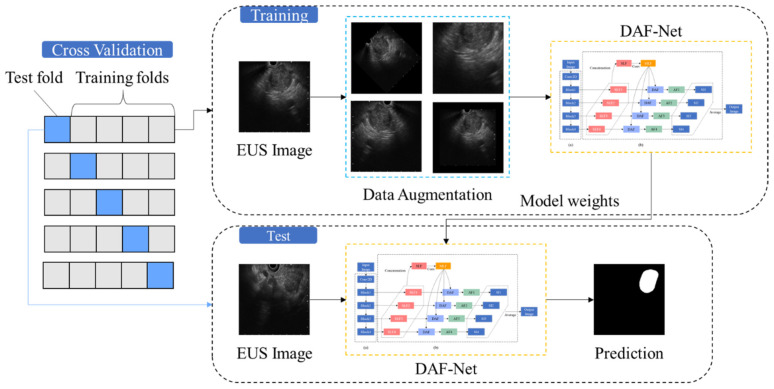
Overall flowchart of the proposed deep learning approach.

Figure 1 shows a flowchart of the proposed deep learning approach for pancreatic cancer segmentation. The acquired EUS data were subjected to five-fold cross-validation.

### 2.3. Data Preparation and Preprocessing

The ground truth (GT) for the segmentation of pancreatic cancer in the EUS images was generated by the endosonographers who determined the cancer area and non-cancer area. For the augmentation of the training data, images were processed by rotating −5° to 5° in steps of 2.5°, scaling by 0.9 and 1.1 times, horizontally flipping, and elastic deformation (by applying random deformation between −2.048 and 2.048 and Gaussian filtering with a standard deviation of 20.48) [15]. Training and test data were resized to 256 × 256 pixels. For 5-fold cross-validation, each fold was split with 66 images from 30 patients.

### 2.4. Deep Learning Approach

**Figure 2:**
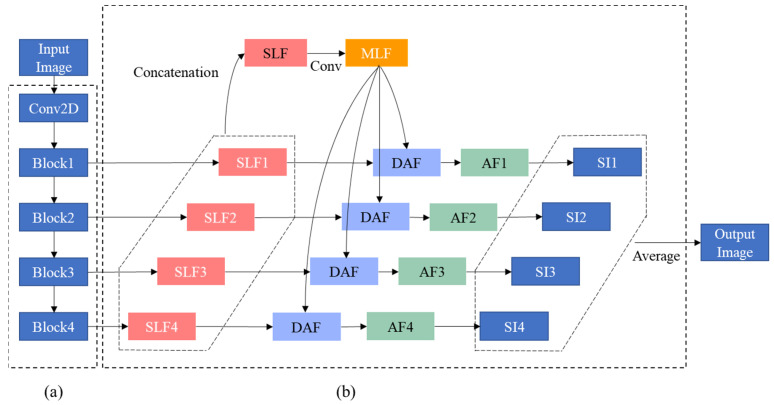
Block diagram of (**a**) encoder and (**b**) decoder parts of DAF-Net.

**Figure 3:**
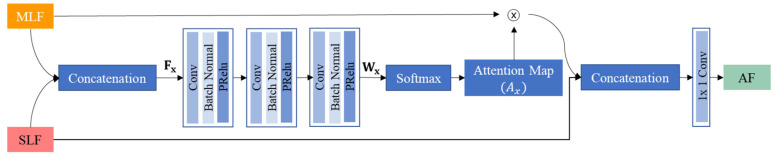
Block diagram of the DAF module.

The deep learning model used in this study is a network with deep attentional features (DAF), which has been proposed for prostate segmentation of transrectal ultrasound images [16] as transrectal ultrasound images are geometrically similar to the EUS images. In this paper, this network is denoted as DAF-Net. DAF-Net contains encoder (Figure 2a) and decoder (Figure 2b) parts. The encoder part consists of ResNext101 [17] and the decoder part consists of single-layer features (SLFs), multi-layer features (MLF), DAF module, attentional features (AFs), and single images (SIs). SLFs are obtained by the linear interpolation of blocks from the encoder part (Figure 2a). An MLF is created by the convolution of a concatenated SLF. Attentional features (AFs) are obtained by feeding SLFs and MLF into the DAF module (Figure 3). SIs are obtained by the linear interpolation of AFs. The output is the mean of the SIs. As shown in Figure 2, the DAF module was used to obtain the AFs. In the DAF module, Fx is obtained by concatenating the SLF and MLF. Then, Fx goes through the convolution block to obtain Wx. Through softmax, the attention map (Ax) is calculated. To acquire the AFs, pixel-wise multiplication of Ax and the MLF is concatenated with the SLF and is followed by a 1 × 1 convolution layer.

The encoder part of DAF-Net was initialized by pretrained weights using the ImageNet [18] dataset, and the decoder part of the network was initialized by random values in the range of [0, 1]. A stochastic gradient descent (SGD) with a momentum of 0.9 and weight decay of 0.01 was used to train the network. The initial learning rate was set to 0.007. The learning rate was reduced by 0.02 times at the 5th epoch and by 0.02 times at the 20th epoch. The network was trained by a batch size of 32 and epoch of 50.

The Dice loss (L) is defined as follows:

(1) $$L=1-\frac{2*n\left(X\cap Y\right)+\mathrm{smooth}\;\mathrm{factor}}{n\left(X\right)+n\left(Y\right)+\mathrm{smooth}\;\mathrm{factor}}$$

where n() is the total pixels in the area, X is the GT area, Y is the segmentation prediction area, and the smooth factor was 1. The total loss L_total_ was determined as the sum of the Dice losses over all the predicted SLFs and SIs:

(2) $$L_{\mathrm{total}}=\overset{k_{1}}{\underset{i\;=1}{\sum }}L_{i}+\overset{k_{2}}{\underset{j\;=1}{\sum }}L_{j}$$

where L_i_ represents the Dice loss of the i-th layer of the SLFs, L_j_ represents the Dice loss of the j-th layer of the SIs, and k_1_ and k_2_ are the numbers of SLFs and SIs, respectively. In this study, *k*_1_ and *k*_2_ are 4. The network was trained and tested using an Intel(R) Core (TM) i9-10900X CPU @ 3.70GHz processor, a CUDA-enabled Nvidia RTX 3090 graphical processing unit (GPU), Python 3.8, and PyTorch 1.10.

### 2.5. Performance Evaluation

The performance of the segmentation results was evaluated using 10 evaluation indices: Dice similarity coefficient (DSC) [19,20], intersection over union (IoU), sensitivity (SEN), specificity (SP), precision (PC), average distance (AVD) [21], mean absolute distance (MAD) [22], Hausdorff distance (HD) [23], receiver operating characteristic (ROC) curve, and area under the curve (AUC). All the values were averaged for 5-fold.

The DSC and IoU are metrics that determine how much the GT and prediction overlap. The two metrics range from 0 to 1, and are defined as follows:

(3) $$\mathrm{DSC}=\frac{2*n\left(X\cap Y\right)}{n\left(X\right)+n\left(Y\right)},\;\mathrm{IoU}=\;\frac{n\left(X\cap Y\right)}{n\left(X\cup Y\right)}$$

where n() is the total pixels in the area, X is the GT area, and Y is the segmentation prediction area. The AVD, HD, and MAD are metrics that measure the distance between a point on the contour of the GT and a point on the contour of the prediction. They are calculated as follows:

(4) $$\mathrm{AVD}=\max\left(\frac{1}{N_{a}}\sum_{a\in A}\frac{\min}{b\in B}d\left(a,b\right),\frac{1}{N_{b}}\sum_{b\in B}\frac{\min}{a\in A}d\left(b,a\right)\right)$$

(5) $$\mathrm{HD}=\max\left(\frac{\max}{a\in A}\frac{\min}{b\in B}d\left(a,b\right),\frac{\max}{b\in B}\frac{\min}{a\in A}d\left(b,a\right)\right)$$

(6) $$\mathrm{MAD}=\frac{1}{2}*\left(\frac{1}{N_{a}}\sum_{a\in A}\frac{\min}{b\in B}d\left(a,b\right),\frac{1}{N_{b}}\sum_{b\in B}\frac{\min}{a\in A}d\left(b,a\right)\right)$$

where A and B represent the contour regions of the GT and prediction, respectively, a and b represent points in the region A and B, d(a, b) represents the distance from a to b, and N_a_ and N_b_ represent the numbers of a and b in the regions A and B, respectively. SEN, SP, and PC are calculated as follows:

(7) $$\mathrm{SEN}=\frac{\mathrm{TP}}{\mathrm{TP}+\mathrm{FN}},\;\mathrm{SP}=\;\frac{\mathrm{TN}}{\mathrm{FP}+\mathrm{TN}},\;\mathrm{PC}=\frac{\mathrm{TP}}{\mathrm{TP}+\mathrm{FP}}$$

which are associated with the true positive (TP), true negative (TN), false positive (FP), and false negative (FN).

### 2.6. Statistical Analysis

To determine if the data were normally distributed, a test was performed using the Shapiro–Wilk test [24]. The Kruskal–Wallis test [25] was used to analyze the results with regard to the T-stage and cancer location. A *p* value greater than 0.05 was considered statistically nonsignificant.

## 3. Results

### 3.1. Deep Learning Network

**Figure 4:**
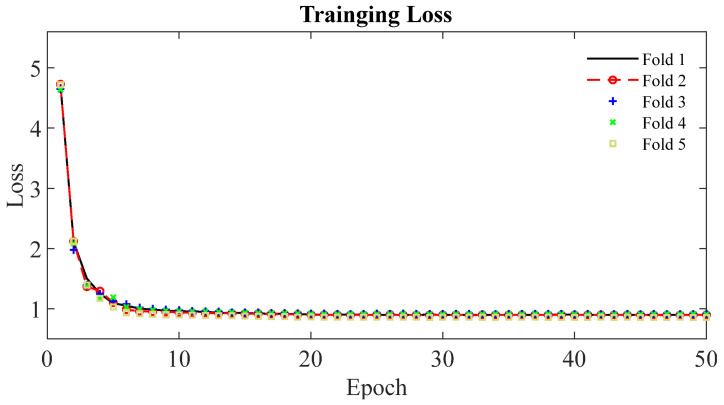
Learning curves of DAF-Net for the 5-fold cross-validation.

Figure 4 shows the learning curves for the 5-fold cross-validation during training of the DAF-Net using pancreas EUS images. It is observed that the learning curves of the 5 folds show similar trends and are flattened at the loss of 0.90 after 20 epochs. Thus, the learning curves show that the network was trained successfully for the segmentation of pancreas cancer using EUS images. The training loss of the DAF-Net is relatively large (>0.9) because the loss (Equation (2)) was computed by comparing the SLFs (Figure 2b) and GTs. SLFs correspond to the upper attention part of the network which is far from the output of the network.

### 3.2. Performance Evaluation

The evaluation results of U-Net, Att-U-Net, and DAF-Net for the segmentation of pancreatic cancer are shown in Table 2. Overall, DAF-Net achieved a higher DSC, IoU, and SEN than U-Net and Att-U-Net. Regarding the distance-based metrics (i.e., AVD, MAD, and HD), DAF-Net performs the best compared to the other networks.

**Figure 5:**
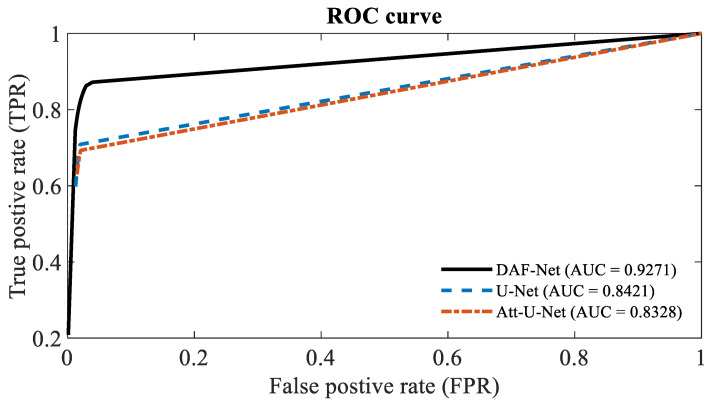
ROC curves for the three different networks (U-Net, Att-U-Net, and DAF-Net).

The ROC curves of U-Net, Att-U-Net, and DAF-Net are shown in Figure 5. The ROC curve of DAF-Net is located more towards the upper-left corner compared with other networks. Also, the AUC for DAF-Net is 10.1% and 11.3% greater than that for U-Net and Att-U-Net, respectively. This implies that DAF-Net is superior to other networks in distinguishing cancerous and non-cancerous regions.

**Figure 6:**
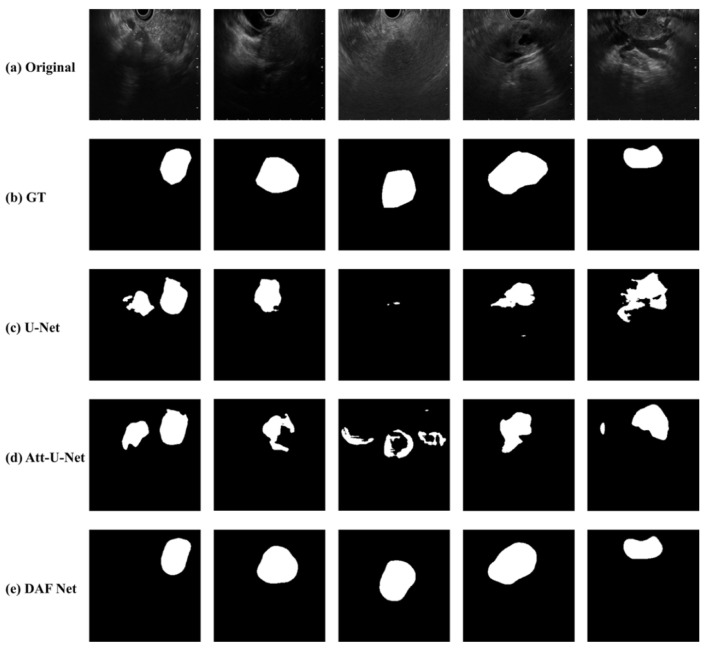
Examples of (**a**) original EUS images with pancreatic cancer, (**b**) GT, and segmentation results from (**c**) U-Net, (**d**) Att-U-Net, and (**e**) DAF-Net.

In Figure 6, examples of original EUS images (Figure 6a), ground truth (Figure 6b), and segmentation results from DAF-Net, U-Net and Att-U-Net (Figure 6c–e, respectively) are shown. While the segmented lesions from DAF-Net are very close to those of the ground truth, U-Net and Att-U-Net cannot predict the shapes of the lesions correctly. In addition, U-Net and Att-U-Net resulted in multiple segmented lesions, although the training was performed with EUS images with a single lesion. Thus, it is clearly observed that, in terms of the quality of the segmentation maps of the DAF-Net, they contain fewer errors and produce more precise segmentation compared to other models. The results match well with the performance evaluation presented in Table 2.

**Figure 7:**
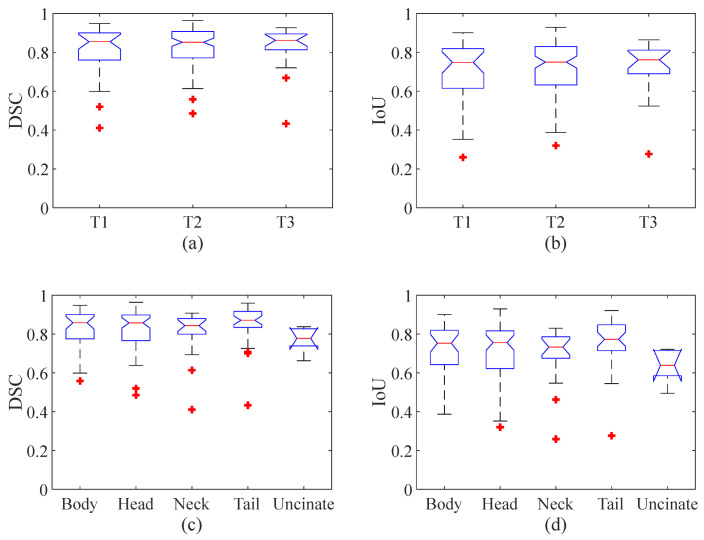
Statistical analysis for the (**a**) DSC and (**b**) IoU for three different cancer stages and the (**c**) DSC and (**d**) IoU for five different locations of the pancreatic cancer. (A red line inside the box represents the median value, the top and bottom whiskers represent the min and max of nonoutliers, respectively, and a red star indicates outliers.).

The segmentation performance in terms of the size and position of the pancreatic cancer were compared by Kruskal–Wallis test and visualized by a box plot. The Kruskal–Wallis tests show that no significant differences (*p* value = 0.98 and 0.9 for DSC and IoU, respectively) were found among the three different stages (i.e., T1, T2, and T3), and that no significant differences (*p* value = 0.13 and 0.13 for DSC and IoU respectively) were found among the five different parts (i.e., body, head, neck, tail, and uncinate). However, the *p* value is relatively low, and the most significant difference was observed between the cancers located in the tail and uncinate where the *p* value was 0.01 for both the DSC and IoU. Figure 7a,b show box plots with the median (red line inside the box), upper and lower quartiles (top and bottom edges of the box), and min and max of nonoutliers (whiskers) of the DSC (Figure 7a) and IoU (Figure 7b) of DAF-Net from three different cancer stages (T1–3), respectively. Figure 7c,d show the median, upper and lower quartiles, and min and max of nonoutliers of the DSC (Figure 7c) and IoU (Figure 7d) of DAF-Net for five different locations of the pancreatic cancer, respectively. Similar to the result from the Kruskal–Wallis test, the box plots show that there is no significant difference in terms of the tumor stage and the position of the pancreatic cancer.

## 4. Discussion

In this study, we conducted pancreatic cancer segmentation using EUS images through a deep learning model. The occurrence of pancreatic cancer is relatively low, and it is more challenging to collect EUS images with the diagnosis of pancreatic cancer than endoscopic images [26]. Thus, the number of studies using EUS images of pancreatic cancer is fewer than those using endoscopy images. Although this study was performed with a limited number of images, overall, the proposed deep learning approach using DAF-Net achieved good performance (>82% DSC and >72% IoU) for pancreatic cancer segmentation using EUS images. Although the performance may vary slightly with the selection of hyperparameters, each network was optimized with the hyperparameters resulting in the best possible performance.

Most of the effort before the surgical therapy of pancreatic cancer is devoted to the staging of the cancer, which is based on the cancer size and location, to avoid misclassification of the resectable area [27]. Due to the surrounding non-cancerous lesions, the boundary of a pancreatic cancer is obscure. Additionally, the presence of arteries and ducts near the pancreatic cancer is the main factor contributing to the uncertainty when segmenting the cancerous region. The pancreas is situated in a very complex position near the liver, kidney, duodenum, and various vascular structures, affecting the ability to clearly define the boundary of the pancreatic cancer. Considering the aforementioned difficulties, there is no single imaging modality that can clearly determine the stage of pancreatic cancer [28]. Because EUS has high operator dependency, it requires a considerable level of experience and competency to stage pancreatic cancer [29]. Hence, the significance of this study lies in the successful segmentation performance of the pancreatic cancer, regardless of the size and location of the cancer, and its usefulness in preoperative planning.

**Figure 8:**
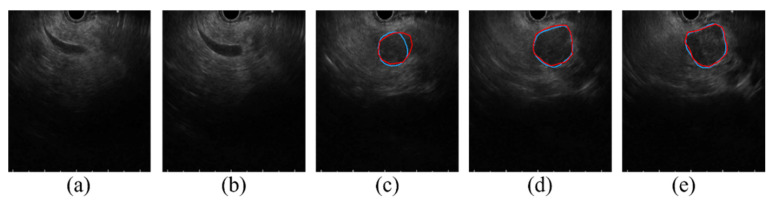
Consecutive EUS images in 0.5 s intervals from a recorded EUS video. (**a**,**b**) show common bile duct with no visible cancerous region, (**c**–**e**) show pancreatic cancer precisely segmented using DAF-Net (Red line: GT boundary, blue line: predicted boundary).

Before the segmentation process, determining whether there is the presence of cancer in the EUS image or not is an important process. Figure 8 shows consecutive EUS images acquired from a recorded EUS video file. Initially, the proposed approach forms no segmentation mask from the EUS images in Figure 8a,b, which show common bile duct without relevance to cancer; then, it forms the segmentation mask to locate the cancerous region in Figure 8c–e. Although the proposed deep learning approach is not developed for the purpose of cancer detection, it can further be extended to determine the presence of the cancer. When the dataset acquired from various situations (e.g., varying scanning locations and angles) is sufficient, our study will be further extended to determine the presence of cancer in the EUS images.

**Figure 9:**
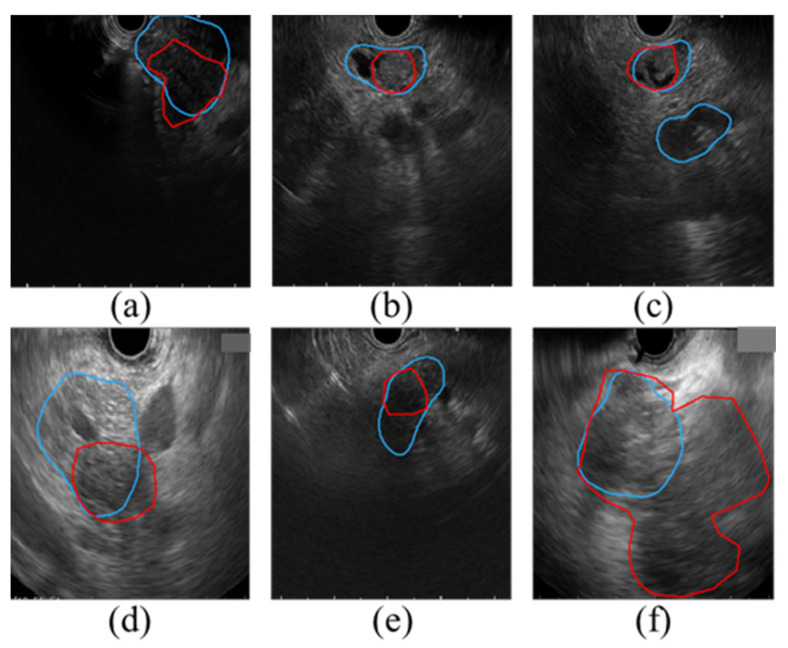
Cases where the IoU is less than 0.5 in the segmentation using DAF-Net (Red line: GT boundary, blue line: predicted boundary). (**a**) EUS probe not properly contacted with the tissue, (**b**) cancer along with pancreatic duct dilation, (**c**) similar texture of the surrounding blood vessels and organs, (**d**) parenchymal atrophy by chronic pancreatitis (**e**) intratumoral necrosis associated with chronic pancreatitis, and (**f**) large tumor size.

To determine the success of the segmentation results, the IoU is commonly used by checking whether the IoU between the segmented area and the ground truth is greater than a predefined threshold value (0.5) [30]. U-Net, Att-U-Net, and DAF-Net achieved an IoU greater than 0.5 in 75.2%, 70.9%, and 90.9%, respectively. While this result shows that the suggested approach can overcome many difficulties in the segmentation of the pancreatic cancer, Figure 9 shows the cases where the IoU is less than 0.5 from the test using our DAF-Net. Figure 9a shows the case in which the EUS probe was not properly in contact with the tissue; thus, the EUS image does not show all the 180 angles of the pancreas, resulting in inaccurate segmentation results. Figure 9b shows a case with pancreatic duct dilation, which was misrecognized as a pancreatic cancer by DAF-Net. In Figure 9c, due to the similarity of the surrounding blood vessels and organs to the pancreatic cancer in the EUS images, other parts were also segmented as pancreatic cancer. As shown in Figure 9d, due to high invasiveness or the accompanying parenchymal atrophy caused by chronic pancreatitis, it is difficult to define clear boundaries of the tumor. In Figure 9e, inaccurate prediction due to intratumoral necrosis associated with chronic pancreatitis is observed. Figure 9f shows that when the size of the tumor is large (5.5 cm in this example), ultrasound penetration can be limited, and a heterogeneous echo is generated due to necrosis, calcified lesions, and degenerative changes inside the mass. Thus, it is difficult to clearly distinguish the main lesion from the marginal area. In the statistical analysis (Figure 7), it was observed that there was no significant difference in the segmentation performance in terms of the size of the tumor and the position of the pancreatic cancer. However, the amount of patient data with cancers in stage T3 or cancers located in the uncinate is relatively lower than those with other conditions (Table 1). Thus, further investigation with more data is necessary. In a future study, more training images from cases that are challenging and include various stages of pancreatic cancer will be acquired to further improve the performance of the current network and confirm the statistical analysis.

The process of pancreatic cancer segmentation using EUS images has several limitations to overcome. First, EUS images can be degraded due to inherent characteristics such as speckles, shadows, and missing boundaries [31]. Thus, contrast-enhanced EUS was utilized to enhance the image quality in other studies [12,32]. By injecting the contrast agent (e.g., microbubbles), one could overcome the weakness of EUS with higher contrast and spatial resolution. However, there still remains the issue of safety when using contrast agents. Despite the inherent characteristics of the EUS images without the contrast agent, the deep learning approach proposed in this study performed well in the segmentation of the pancreatic cancer. Second, there are two types of EUS, which are radial EUS and linear EUS. In this study, images from linear EUS were used only to train and test the deep learning network. For future work, images from radial EUS will also be utilized. The accurate and precise segmentation of the cancer area is critical for the surgical therapy of pancreatic cancer. In addition, vascular invasion is an important factor to diagnose the stage of cancer and plan for the therapeutic strategy [33,34]. Thus, our future work will include an investigation of vascular invasion in pancreatic cancer using a deep learning approach. Therefore, the proposed method in this study can be improved to help the diagnosis as well as the decision for the therapy to treat pancreatic cancer.

## 5. Conclusions

In summary, the proposed deep learning approach using DAF-Net provides a superior segmentation performance for pancreatic cancer using EUS images. It is expected that the proposed accurate and precise segmentation approach can provide effective and efficient assistance in planning for the surgical therapy of pancreatic cancer.

## Figures and Tables

**Table 1:** Characteristics of the patients and cancer.

Characteristics	Count
Age, years, mean (range)	71.2 (40–94)
Gender (male/female)	96/54
Cancer size, mm, mean (range)	31.0 (10–130)
Cancer T-stage (T1, T2, T3) [9]	33/99/18
Cancer location (body, head, neck, tail, uncinate)	37/60/18/33/6

**Table 2:** Summary of network performance evaluation (Bold font indicates best result obtained).

Method	DSC	IoU	AVD	HD	MAD	SEN	SP	PC
U-Net	0.74	0.62	13.84	44.26	10.47	0.80	**0.98**	0.83
Att-U-Net	0.72	0.60	14.69	43.47	11.28	0.78	0.98	0.81
DAF-Net	**0.83**	**0.72**	**9.04**	**27.35**	**7.53**	**0.84**	0.98	**0.85**

## Data Availability

The data presented in this study are available on request from the corresponding author.
